# Probability and Neurodegeneration: Alzheimer’s Disease and Huntington’s Disease

**DOI:** 10.3390/brainsci15080814

**Published:** 2025-07-29

**Authors:** Peter K. Panegyres

**Affiliations:** 1 Neurodegenerative Disorders Research Pty Ltd., Perth, WA 6005, Australia; research@ndr.org.au; Tel.: +61-8-6317-9472; 2 School of Medicine, The University of Western Australia, Perth, WA 6009, Australia

**Keywords:** probabilistic determination, young-onset dementia, Alzheimer’s disease, Huntington’s disease, somatic mutations

## Abstract

Background: The mechanisms by which sporadic young-onset neurodegenerative processes develop are uncertain. Methods: We have previously proposed that stochastic processes involving sequence changes at a DNA, RNA, or protein level in critical genes and proteins might be important to this process. Further investigation points to the contribution of probabilistic states in other factors involved in neurodegenerative conditions, such as—in the case of young onset Alzheimer’s disease—head injury, apolipoprotein ε4 alleles and other elements that, by the interaction of conditional probabilities in these variables, influence the evolution of neurodegenerative conditions. Results: This proposal might help to explain why some autosomal dominant neurodegenerative conditions, such as trinucleotide repeat disorder (Huntington’s disease), might have variable ages of onset given the same disease-causing CAG repeat mutation length. Conclusions: The detection of somatic mutations in single brain cells provides some experimental support for these emerging concepts.

## 1. Introduction

It has come to our attention that probabilistic mechanisms may play an important role in the pathogenesis of neurodegenerative processes, particularly as they pertain to the development of sporadic neurodegenerative disorders in young adults, especially young-onset dementia [1,2].

Earlier, we theorized that probability determined sequence changes in critical DNA, RNA, or protein molecules trigger the neurodegenerative pathways and declared the evolutionary significance of such probabilistic mechanisms [2,3]. Here, we posit that other fields within biological probability space influence the advance of neurodegenerative disorders.

Consider the neurological problems that we face daily in clinical work: a 50-year-old man, along with his family, reported his progressive episodic memory decline. An MRI showed mesial temporal atrophy. A neuropsychological assessment confirmed episodic memory abnormalities, with additional findings of impaired attention, orientation, linguistic function, visuospatial abilities and mathematical and praxic skills. A fluorodeoxyglucose positron emission tomography (FDG-PET) scan revealed reduced metabolism in the temporal-parietal networks and in the precuneus region, which is consistent with Alzheimer’s disease (AD). The brain amyloid PET scan revealed an abundance of amyloid protein, confirming the clinical diagnosis of AD. This man, a typical patient in our young-onset dementia clinics, had no history of head injury, no hypertension and no diabetes mellitus or other cerebrovascular risk factors. He did not consume alcohol, cigarettes, or illicit drugs and did not have a family history of AD or other neurodegenerative conditions. He was well educated, with a university degree in physics and worked as a high school teacher. He was married, with two young children and reported no obvious psychosocial stressors. He ate a predominantly Mediterranean diet and exercised at least five times per week in a combination of aerobic and resistance activities, for about 45 min each session. He did not possess gene mutations involving the amyloid precursor protein (APP) gene or presenilin 1 and 2 (PS1 & 2), which are associated with young-onset AD. He was APOE ε4 2/3. Why did this man, without any obvious risk factors, develop AD? We posit that stochastic and probabilistic elements have propelled this process. This article puts forward a hypothesis as to how.

## 2. Materials and Methods

### 2.1. The Mechanisms

Previously, we theorized that the probability of a neurodegenerative process occurring in anatomical space was determined by the equation of probabilistic sequence changes in DNA, RNA and proteins [2]:

$$P_{AS}\;\left(neurodeg\right)=f(P_{DNA},\;P_{RNA},P_{PROTEIN})$$

$$P_{AS}\;=\;\mathrm{Probability}\;\mathrm{of}\;\mathrm{neurodegeneration}\;\mathrm{in}\;\mathrm{an}\;\mathrm{anatomical}\;\mathrm{space}$$

We propose that a single deviant molecule can steer the production of other molecules, leading to pathological overabundance and disease in anatomical space:

$${PS}_{ND}\to {PS}_{ND1}+{PS}_{ND2}+{PS}_{ND3}+\cdots {PS}_{NDX}$$

PS = Protein vector in anatomical space

Here, we refine and expand this model to reflect the contribution and mechanism by which other factors play a part in this process. Whether these processes evolve into neurodegenerative disorders depends on the probabilistic state of other variables (Table 1).

Thus, our probability function, $P_{AS}\;\left(neurodeg\right)$, can be extended to include these factors:

$$P_{AS}\left(neurodeg\right)=f(P_{DNA},P_{RNA},P_{PROTEIN},A,Β,\Gamma ,\Delta ,Ε,Ζ,H,\Theta ,Ι,Κ,\Lambda )$$

If we take the presence of a gene mutation in presenilin 1, representing the most common cause of genetically determined AD, the probability of a neurodegenerative process occurring, given the presence of a gene mutation in PS1, approaches 1:

$$P_{AS}(neurodegen)\to 1\;(99.9\%)$$

The conditional probability of $P_{AS}\;\left(neurodegen\right)$, based on these other factors influencing the development of AD and in the absence of autosomal dominant gene mutations, is much less; the exact probability for each variable is unknown, and the probabilistic state is likely to be unique to the individual. The exact probability for these factors might be impossible to determine in each patient (e.g., the role of deafness) until the probabilistic nature of the other variables is realized; that is, the uncertainty in each patient is such that $0\leq P_{AS}\;(neurodegen)\leq 1$. So then, how does a patient without obvious risk factors or gene mutation develop AD? It is postulated in our clinical example above that, possibly:

$$P_{AS}\;\left(neurodegen\right)[our\;patient\;with\;YOAD]=f\left(P_{DNA},P_{RNA},P_{PROTEIN},Κ,\Lambda \right)$$

$$P_{AS}\;\left(neurodegen\right)[other\;patients\;with\;YOAD]=f\left(P_{DNA},P_{RNA},P_{PROTEIN},\Gamma ,\Delta \right)$$

Many permutations are possible, and it is likely that the combination may be different for each patient, with the same diagnostic outcome. That is, different probabilistic combinations of f → the same diagnostic outcome and could activate a similar and identical pathological reaction; that is, the amyloid cascade acts as the final pathophysiological pathway. The conditional probabilities of an outcome of AD are based on one or more of the variables listed—clearly, not all variables are equal, and some must have greater effects than others. (The determination of the exact degree of contribution of each variable is the subject of ongoing research.)

**Figure 1:**
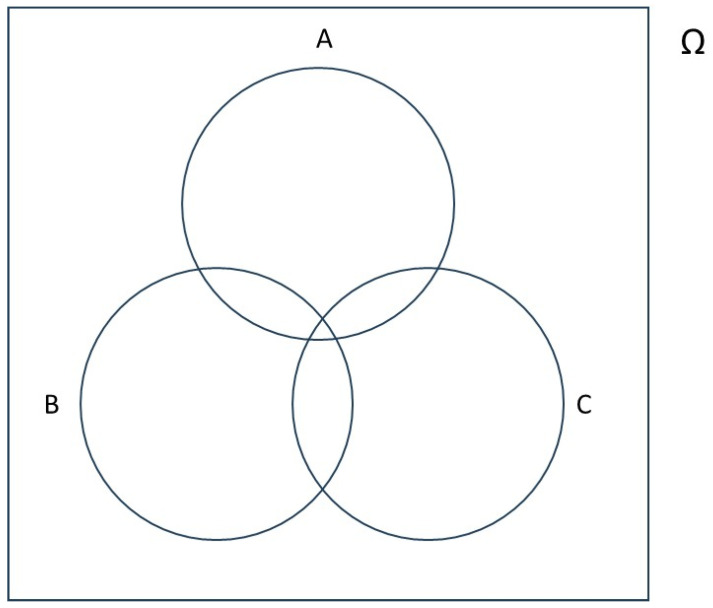
Venn diagram representing a scenario with two risk factors for the development of Alzheimer’s disease.

A visualization of the conditional probabilities for a scenario involving two risk factors, 1 and 2, is possible using a 3-way Venn diagram (Figure 1). In the diagram below:

$$A=\left(population\;with\;YOAD\right),$$

$$B=\left(population\;with\;risk\;factor\;1\right),$$

$$C=\left(population\;with\;risk\;factor\;2\right),$$

$$\Omega =\left(general\;population\right).$$

Thus, it follows that:

$$P\left(A|B\right)=\frac{P(A\cap B)}{P(B)}=\frac{\left|A\cap B\right|}{\left|B\right|}$$

$$P\left(A|C\right)=\frac{P(A\cap C)}{P(C)}=\frac{\left|A\cap C\right|}{\left|C\right|}$$

$$P\left(A|B\cap C\right)=\frac{P(A\cap B\cap C)}{P(B\cap C)}=\frac{\left|A\cap B\cap C\right|}{\left|B\cap C\right|}$$

The notation P(A|B) represents the probability of A given B, $P\left(A|C\right)$ is the probability of A, given C, and $P\left(A|B\cap C\right)$ is the probability of A given both B and C.

The notation |A| represents the number of people in set A, |*B*| is the number of people in set B and |*C*| is the number of people in set C. |A∩B| is the number of people common to both sets A and B. |B∩C| is the number of people common to both sets B and C. |A∩B∩C| is the number of people common to all three sets.

Reducing these probabilities to ratios of numbers allows the probabilities to be calculated empirically.

Therefore, the probability of developing a neurodegenerative process is a result of the probabilistic state of the other variables. The probability of developing young-onset AD (YOAD) is low, and the standardized prevalence of YOAD is approximately 119/100,000 population [4], in comparison to old-onset AD (i.e., onset after the age of 65 years). One in thirteen people aged 65–84 years will develop AD, in comparison to one in three people over the age of 85 years [5]. The probability of developing YOAD is not zero, and the probability of AD increases with age.

That is, the probability of developing YOAD is dependent on the probabilistic states of other neurodegenerative variables and suggests that the process can be analyzed using conditional probabilities; that is, YOAD and, by extension, other neurodegenerative processes are probabilistically determined. We prioritize the development of clinical protocols and decision support systems to develop the concepts presented herein.

**Figure 2:**
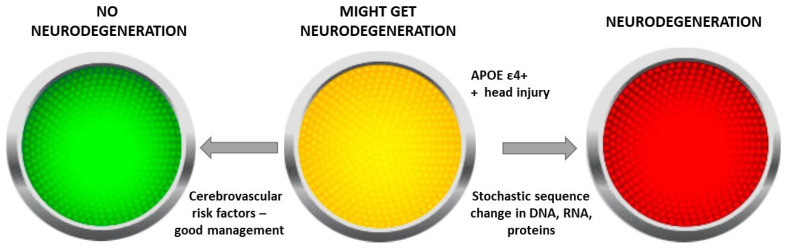
Traffic light model of the probabilistic determination of neurodegenerative disorders through the example of young-onset dementia.

We suggest that this hypothesis may be illustrated by a traffic light model (Figure 2). The light is red for neurodegeneration in the context of AD-causing mutations, such as the PS1 mutation, which will lead to over 99% probability of YOAD (Figure 2, red light).

**Figure 3:**
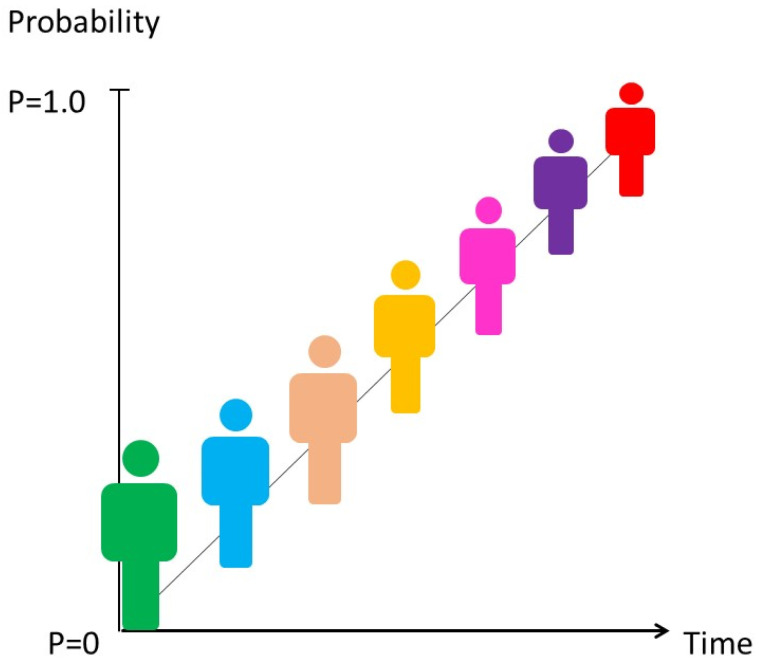
Humans exist in a probability continuum for the development of neurodegenerative disorders.

If a person has no risk factors, the probability is very low and approaches zero; that is, the light will be green, i.e., not resulting in neurodegeneration. A person in the amber zone may have an increased risk of YOAD due to the presence of, say, significant cerebrovascular risk factors or multiple head injuries in the context of the presence of an apolipoprotein ε4 allele, which will then propel them into YOAD. This model permits a person who is in the amber zone to improve their risk status, through good management of cerebrovascular risk factors, control of deafness and lifestyle modifications, and move to the low-risk green zone (Figure 2). This model supports the notion that AD, and possibly the risk of neurodegenerative disorders in general, is modifiable. Furthermore, the model suggests that humans exist in different states of probability of developing a neurodegenerative disorder like AD—some are at low risk, others at high risk and some intermediate. Some risk factors are modifiable and can influence the probability of transformation to neurodegeneration determined by time (Figure 3) [6]. In our Alzheimer’s Prevention Clinic, we emphasize and manage modifiable risk factors in our vulnerable patients.

### 2.2. Probability in Neurology

Probability is fundamental to biological processes and is essential to the organization of biological systems and adaptation [7,8,9]. Probability determines genotypes with greater survival likelihood, depending on environmental pressures, and is the foundation of homeostatic mechanisms, which promote survival and selection [10,11,12,13]. Probability drives the molecular bases of evolution [14,15].

Indeed, probability is considered the foundation of the evolution of life when dealing with environmental uncertainty [16]. The increase in brain size in comparison to body size over millions of years suggests that selection pressure originates in the brain and that probabilistic and stochastic alterations in DNA, RNA, or proteins are the molecular basis of this evolutionary process and are basic to human survival as a species [17]. Probabilities might also determine cognitive processes that also contribute to probabilistic events, and this is relevant to our investigation, in which lifestyle, diet, the prevention of deafness and other factors affect the development of neurodegeneration [8]. Probabilistic processes have been suggested in signaling networks and synthetic biological systems [18,19,20]. Modeling using Bayesian linear mixed effects to investigate the path of change in AD and frontotemporal dementia reveals that accelerated atrophy in the mesial temporal structures, insula, frontal, anterior and inferior frontal regions over two MRI scans predicted conversion to dementia and, in other studies, the rate of atrophy in the hippocampus and ventricular volume [21,22]. Bayesian models can be powerful if using robust data—like brain scans—and can account for fixed effects (e.g., age, genetics and cognition) and random effects (e.g., individual variations in the rate of brain atrophy). Bayesian modeling allows for non-linear dynamics in variables like brain atrophy and cognitive decline. However, not all variables are quantifiable (e.g., deafness and biophysical parameters), limiting the application of Bayesian inference and mixed-effects modelling, compounded by disease heterogeneity, an incomplete comprehension of disease pathophysiology and individual variation.

Quantum probability, in its application to open biological systems, involves information processing in complex biological systems and their interactions with their environment. Such investigations will elaborate the contribution of quantum-like modeling to neurodegenerative operations at molecular and sub-molecular levels [23,24]. Neurodegenerative disorders result from protein misfolding and from molecular and cellular interactions. Protein folding involves quantum effects, including electron distribution, superposition and quantum interference in three-dimensional space; electron transfer in processes like oxidative stress—which is important in neurodegenerative disorders—may be influenced by the behavior of electrons at a molecular level, electron transfer and quantum tunnelling in neurons and glial cells, probably in the mechanisms of cell death and neurodegeneration. Classical probability models are useful at a population level but do not help to explain molecular differences in disease onset and progression, where quantum tunnelling and quantum interference possibly determine protein folding.

### 2.3. Experimental Verification

Advances in neurosurgical techniques [25], cryo-electron microscopy, in which visual proteomics identifies proteins *in situ* within single cells [26,27], and single-cell biochemistry [28,29] have advanced the concepts that changes in DNA, RNA and protein sequences at a single-cell level may be a fundamental biological mechanism in sporadic neurodegenerative disorders and other neurological conditions, including focal cortical dysplasia, hemimegalencephaly, temporal lobe epilepsy and arteriovenous malformations [25,30,31,32,33]. A study of over 100 brains revealed that 6% of brains had somatic variations at a DNA, RNA and protein sequence level in normals and in people with Tourette’s syndrome, schizophrenia and autism—such a sequence variation was also associated with age and cancer; duplications and *in vivo* clonal expansions were also identified [34,35]. DNA replication errors, environmental exposures, cellular stress and epigenetic changes influence brain cell genetics and cause physico-chemical disorder, resulting in changes in gene expression, embryological activity, synaptic function and the excretion of damaged proteins, all of which determine different types of human brain pathology. Somatic mutations can result in changes to important proteins like Aβ and Tau that influence their folding, assemblage and clearance, which causes AD. These mutations might have differential effects on these proteins, causing some individuals to have rapidly evolving dementia and different patterns of onset (e.g., posterior cortical atrophy vs. the frontal variant of AD). Other pathophysiological stressors—like environmental and cellular exposure—further complicate the pathobiology. Autism spectrum disorders might result from somatic mutations and environmental factors at important stages of embryology that influence brain wiring, thereby compromising sensory circuits and behavior, especially when involving genes with important synaptic functions and neurotransmitters, with consequences from mild functional effects to severe behavioral difficulties. Similarly, Tourette’s syndrome probably arises from random somatic mutations in those genes important for dopamine function in striatal circuitry, causing tics and other movement disorders. These random mutations alter the degrees of symptoms, extent and natural history.

**Figure 4:**
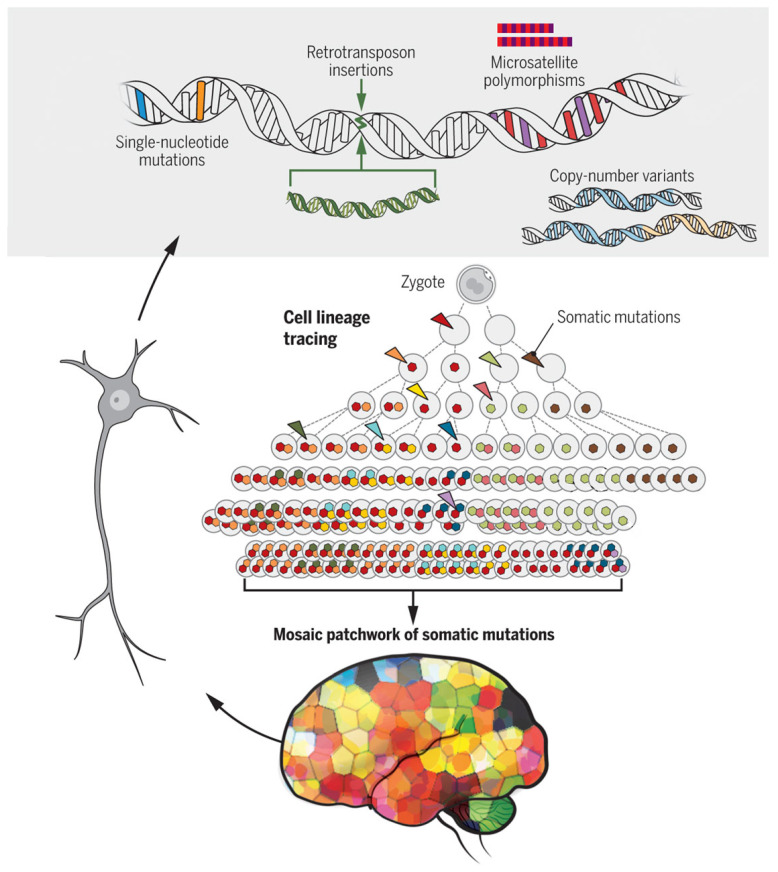
Somatic mutation events. Single-cell genomic techniques enable the systematic measurement of somatic mutations in the brain. Our brains are a patchwork of somatic mutations that increase with time. (Graphic: adapted from G.D. Evrony [36] by K. Sutliff and reproduced with permission from *Science*).

Single-neuron genomics researchers have discovered small point mutations, microsatellite polymorphisms, larger retrotransposon insertions, copy number variants and aneuploidy [28,31,36,37] (Figure 4). These spontaneously occurring somatic mutations originate from stochastic mechanisms in which somatic mutations have anatomical restrictions and might explain localized presentations of neurodegenerative disorders, such as frontal sporadic AD, linguistic forms and posterior cortical atrophy. In fact, some somatic mutations have been found in areas of less than 1 cm^2^ of the human frontal cortex [29,30].

**Figure 5:**
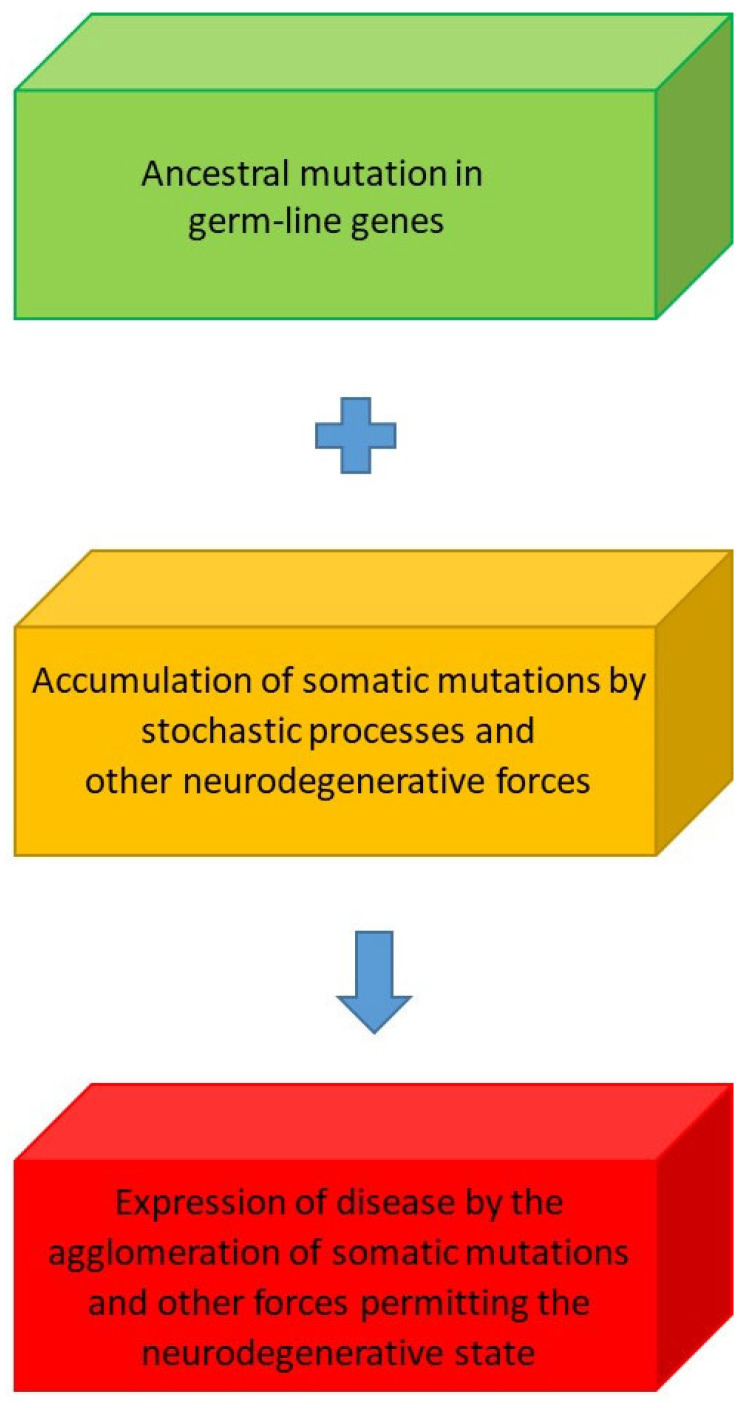
The interaction of germline and somatic mutations and other factors determining the onset of neurodegenerative processes.

Our concept of stochastic processes underlies these somatic mutations and is distinct from mutation in germline cells; it is possible that germline mutations and somatic mutations have a synergistic interaction, which might explain why inherited neurodegenerative disorders like AD, Huntington’s disease (HD), fronto-temporal dementia, genetic forms of prion disease, Parkinson’s disease (PD) and motor neuron disease manifest in later life (Figure 5).

Such a hypothesis might explain why some people with the same CAG repeat length (CAG repeat length of ≥40 causes HD and the pathological expansion of the huntingtin protein) manifest HD at 40 years of age, whereas others over 60 or even 80 years of age do not. People with HD, genetic PD and fronto-temporal dementia (FTD) show different presentations and natural history, which is probably a result of the following: interactions between an inherited genetic factor, in the case of HD, and somatic mutations that disrupt neuronal and glial cell function and occur randomly in space and time; modifying genes that promote or delay the disease; environmental factors (e.g., lifestyle, stress, toxins); and epigenetic factors that may alter gene expression. These forces probably contribute to the phenotypic variations between patients seen in clinical practice.

Recent research suggests that genes for modulating DNA repair and the stability of DNA repeats drive the continuing expansion of CAG repeats to >150, causing toxicity, progressive neuronal death and symptomatology in HD [38,39].

## 3. Results

**Figure 6:**
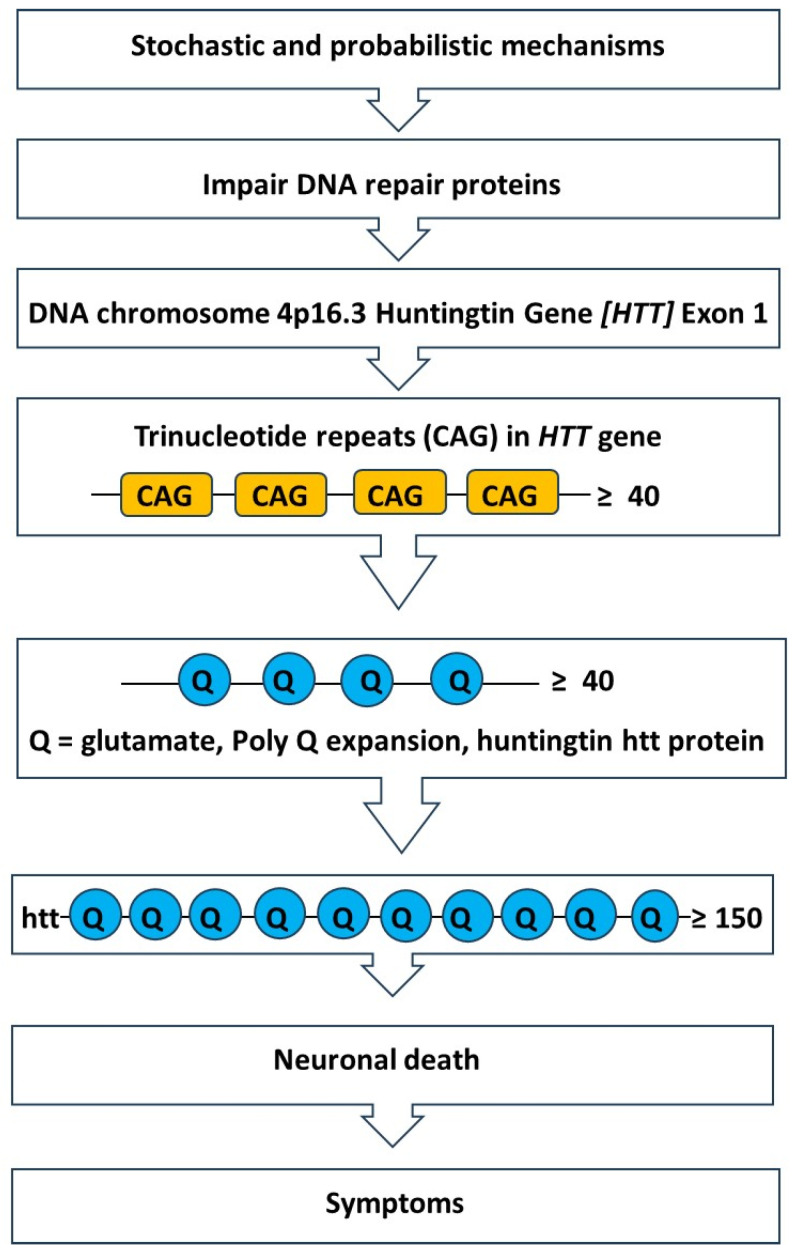
The postulated interaction between stochastic and probabilistic mechanisms, impaired DNA repair proteins and the molecular pathogenesis of Huntington’s disease.

We postulate that the molecular machinery involved in this process undergoes pathological change by the probabilistic mechanisms described herein, and that the clinical heterogeneity observed in HD natural history is powered by stochastic and probabilistic processes. These considerations lead to innovative therapeutic approaches to treating CAG repeat length disorders (HD, spinocerebellar ataxias, etc.) and have implications for the treatment of other neurodegenerative conditions. The challenge is to comprehensively explore these stochastic and probabilistic mechanisms in each neurodegenerative condition (Figure 6).

These single-cell genomic techniques indicate that humans are a composite of somatic mutations generated by stochastic processes, influenced by the large number of physical, biochemical and other variables that influence AD, specifically YOD and other forms of neurodegenerative processes. Maybe these variables might enhance the development of somatic mutations themselves [40,41,42]. Somatic mutations in brain cells, non-germline, result from DNA replication errors, environmental variables and physicochemical stressors. These molecular changes increase with time and adjust the natural history of conditions like HD. The analysis of individual brain cells allows us to elucidate the expression of these mutations at a cellular level, along with their functions and the molecular cellular processes by which neurodegeneration is modified. Detecting these mutations in brain tissue is difficult because of the variety of brain cells that may be distinctively affected by random mutations and distributed in anatomical space, leading to sampling errors: isolating single brains remains a technical problem; neuro-inflammation might obscure mutant cells; obtaining fresh brain tissue is a challenge; and post-mortem sampling—performed at the end of life—might be conducted too late to detect meaningful changes because of general brain disintegration from global mutations.

Somatic mutations have been identified in the α-synuclein gene, where a recombination mechanism resulted in somatic variants of the amyloid precursor protein gene causing AD, with the α-synuclein toxic gain of function effects resulting in PD and multiple system atrophy, single nucleotide variants affecting tau phosphorylation leading to AD and single nucleotide variants in DNA repair genes causing motor neuron disease [41,43].

DNA sequences in blood and brain samples from patients with semantic dementia showed somatic variants in brain tissue but not in blood: two variants in Exon 1 of the transactive response DNA binding protein 43 kDa (TARDBP43) gene in the cerebral cortex and dentate gyrus [44]. These mutations impaired the splicing of TARDBP43 and altered the subcellular localization of the TARDBP43 protein. Somatic mutations have also been found in AD [45].

Harmful somatic gene variants were found in 10% of sampled cells in 1% of brains (N = 146), with C > T variants being most common, indicating DNA mismatch and repair in genes prevalent in the brain [46]. Other studies have found somatic single-cell variants in genes with synaptic functions and neuronal processes and not disease causing known familial PD-causing genes [47].

These findings support the concept that, in certain patients, sporadic neurodegenerative disorders may occur in genes that interact with known germline mutations—such as presenilin 1 in AD and leucine-rich repeat kinase 2 (LRRK2) in PD. That is, the somatic mutation thus identified may not be in known autosomal dominant genes, resulting in recognizable diseases, but in genes causing a protein malfunction that initiates the pathophysiological cascade, leading to AD and other neurodegenerative states, as revealed in HD [48]. More work is required to confirm this hypothesis as experimental verification of somatic gene variants in neurodegenerative disorders is complex [49]. Furthermore, brain somatic mutations may be amenable to an RNA therapeutics approach with antisense oligonucleotides and small interfering RNAs [50]. RNA interference (RNAi) targets the RNA before protein translation, using small interfering RNA (siRNA) or short hairpin RNA (shRNA) that could target damaging proteins to reduce expression and avoid accumulation (e.g., Aβ or Tau). Anti-sense oligonucleotides are short strands of RNA that bind to specific mRNA molecules and influence gene expression, as in spinal muscular atrophy; CRISPR-Cas9 technologies have the potential to edit DNA, specifically somatic mutations. However, RNA technologies may have unintended consequences: off-target effects, turning off other genes that are important for cellular functions; difficulty targeting specific brain cells; intra-thecal administration has risks; lack of tissue substrate sensitivity; triggering an immune response that might worsen neurodegeneration; the formation of new and deleterious mutations; complex pathophysiology may limit application if the pathophysiological pathway is initiated; and the long-term safety of these agents is unknown.

## 4. Discussion

It is set forth elsewhere that probability governs the neurodegenerative processes that determine the development of sporadic young-onset dementia. These aleatory mechanisms are especially relevant to understand the development of AD in people aged 19–28 years, in the absence of recognized gene mutations [51]. Stochastic sequence variation at a DNA, RNA, or protein level in critical genes and proteins can combine with probability states in other neurodegenerative risk factors that, by the interaction of conditional probabilities of these elements, determine the development of variants of AD and other neurodegenerative conditions; that is, the development of sporadic AD and dementia depends on the probabilistic state of a number of interacting variables. This hypothesis could be tested experimentally by further research: single-cell sequencing and mutation profiling on brain samples from different anatomical locations obtained from subjects at post-mortem with neurodegenerative disorders and other conditions; inducing stochastic mutations in experimental animals, using CRISPR-Cas9 methods to induce specific mutations to measure the outcome; studying brain cell aging and mutations in post-mortem samples from people with AD or HD and analyzing single cell sequences to quantify the number of mutations in different anatomical regions over time; exploring clonal expansion in neurons to see if an isolated cell (e.g., brain precursor cell) proliferates and contributes to disease, using genetic barcoding to follow clonal expansion in experimental animals by introducing distinctive mutations with viral vectors or transgenic techniques and monitoring the expansion of clones in different anatomical brain regions over time; studying the influence of environmental factors on mutations in experimental animals; investigating genetic mosaicism in brains collected at necropsy by single-cell sequencing in brain tissue collected from different brain regions and correlating the findings with the clinical phenotype. Such investigations will help unravel the role of stochastic process in neurodegenerative disorders. A traffic light model helps to understand this hypothesis and explains why some patients may shift from a high risk to a low risk and from a low risk to a high risk, depending on their behavior, and that humans are on a probability continuum of developing neurodegenerative disorders like AD. Advances in neurosurgery and single-cell biochemistry provide some proof for this hypothesis by the detection of somatic mutations in autosomal dominant genes known to cause AD, PD and other conditions, and in genes involving DNA repair and neuronal and synaptic function.

Finally, this hypothesis might also explain why people with known gene mutations causing AD or other neurodegenerative conditions, like HD, might have an earlier or later presentation as a result of stochastic and probabilistic mechanisms that allow neurodegeneration. The stochastic mechanisms may be different for each sporadic neurodegenerative process, but the fundamental mechanism of randomly determined sequence changes in key molecules remains essential for sporadic neurodegenerative disorders that are conditional upon probabilistic factors. These stochastic mechanisms are pertinent to autosomally dominant inherited conditions, like HD, which may influence natural history.

## 5. Conclusions

Future work mandates that the contributions of probabilistic states and stochastic variables that influence neurodegenerative conditions need to be determined more precisely and that artificial intelligence (AI), supercomputing and the analysis of quantum effects might help. AI and supercomputing assist in elucidating the role of stochastic processes and probability in neurodegeneration by: analyzing large and intricate datasets; using machine learning to develop models of prediction based on genomic, clinical and imaging information; performing computer simulations and modeling pathophysiological states; the development of personalized medicine to individualize treatments using genetic and clinical data; recognizing new therapeutic targets by analyzing huge datasets of genomic, proteomic and metabolomic data; and detecting environmental variables (e.g., diet or pollution) that, in the presence of genetic predisposition, might determine neurodegeneration. To ensure this progress, data quality and standardization are essential: integrating datasets (e.g., clinical, imaging, genetics and proteomics); ensuring the interpretability of AI models and using techniques like explainable AI to understand predictions; avoiding data bias; model generalization using cross-validation, external validation and continuous learning; ensuring ethical and privacy concerns are addressed, data protection and informed consent; confirming the clinical validation of AI predictions; ensuring adequate computer power and storage for AI, including deep learning and neurodegenerative simulations that require cloud computing; utilizing distributed computing frameworks and more efficient algorithms (edge AI), which will aid in the elucidation of the role of stochastic and probabilistic forces in neurodegeneration. Such validation studies must involve different populations, cultures, environmental factors and lifestyles. This data analysis will help illuminate the role of stochasticity and probability in sporadic and inherited neurodegenerative disorders worldwide and could lead to the discovery of new treatments.

## Figures and Tables

**Table 1:** Probabilistic states and neurodegeneration.

Probabilistic State Designation	Processes
A	Gene mutations (APP, PS1 & 2)
B	Cerebrovascular risk factors (hypertension, stroke, diabetes mellitus, or dyslipidemia)
Γ	APOE ε4/ε4
Δ	Head injury
E	Age
Z	Brain health and lifestyle (education, exercise, smoking, diet, alcohol, body weight, sleep, social network and engagement and cognitive activity)
H	Deafness
Θ	Socioeconomic status
I	Ethnicity
K	Biophysical (microenvironment, pH, immune reactions, glial responses and phagosome function)
Λ	Unknown (yet to be determined)

## Data Availability

The original contributions presented in this study are included in the article. Further inquiries can be directed to the corresponding author.
